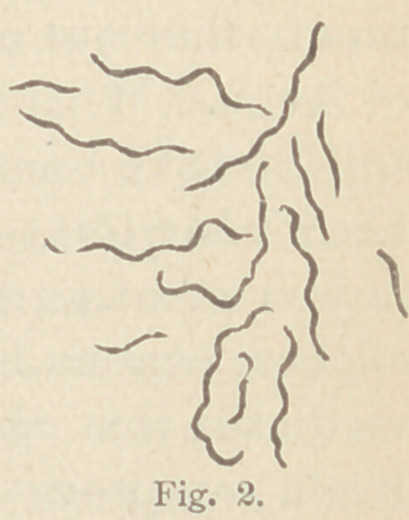# A Comma Bacillus in the Human Mouth

**Published:** 1885-05

**Authors:** W. D. Miller

**Affiliations:** Berlin


### A COMMA BACILLUS IN THE HUMAN MOUTH.

DEMONSTRATED FEB. 16TH, AT THE *Verein Fuer Innere Medicin.*

BY PROF. DR. W. D. MILLER, BERLIN.

It is a well known fact that comma-shaped bacilli are constantly present in the human mouth, even in a state of perfect health. No particular importance was, however, attached to this fact, until these bacilli were, by Prof. Lewis, asserted to be identical with Koch's comma bacilli of Cholera Asiatica. Since that time, very many bacteriologists have constantly endeavored to obtain the comma bacilli of the mouth, in pure culture. In the *Deutsche Medicinische Wochenschrift*, 1884, I described two different schizomycetes which I had isolated from the oral secretions, and which in certain cases appeared as distincly curved bacteria.

Not until within a few days, however, did I succeed in isolating from the mouth a true comma bacillus, and it was finally accomplished, in two cases, by the use of coagulated beef blood serum. The material from which the cultures were made was, in each case, found under the margin of inflamed gums, in unhealthy mouths. Morphologically this bacillus is very similar to the other well known comma bacillus, occurring as commata, either singly or in twos (Fig. 1), or in spirillum form. (Fig. 2.) In old cultures of gelatine, all the commata sometimes grow out into spirilli, giving a pure spirillum culture, cultivated on plates of beefwater-peptone-gelatine, at 20° C. They appear after twenty hours (in the second dilution), under a power of 100 diameters, as perfectly round, finely granular colonies, with a smooth border and brown color; in the same time the first dilution will be completely liquified. They liquify coagulated blood-serum with great energy, as do the other comma bacilli. On the surface of Agar-Agar they formed a yellowish coating, and convert the medium, superficially only, into a paste. They grow slowly on boiled potato. I have, consequently, not yet been able to establish any definite peculiarity of growth. The reactions of this bacillus are such as at once establish the fact that it is altogether a different organism from the comma bacillus of Koch. It possesses, on the other hand, many of the peculiarities of the Finkler-Prior bacillus. Whether it is identical with this organism, must be established by further experiment.

Fig 1.

Fig. 2.

It must be remarked that this organism is, in all probability, not the one which is constantly to be found in every mouth. This grows rapidly on ten per cent. gelatine, while the latter appears to be unable to grow at all on the same medium.

Dr. Klein, in his report on Cholera Asiatica (British Med. Journal, Feb. 7th, 1885), states that the comma bacilli of the human mouth have the same peculiarities of growth on gelatine with the comma bacilli of Koch, but not one of the many forms of micro-organisms, curved or otherwise, which I have obtained in pure culture from the human mouth, is for a moment to be mistaken for the bacillus of Koch. If Dr. Klein has really succeeded in proving by culture methods, that the common comma bacilli (vibriones) of the human mouth have the same reaction on gelatine as Koch's comma bacillus, then it is very desirable that we should be told how he did it, it being the testimony of a very large number of bacteriologists that these organisms do not grow at all on gelatine. An exact proof that the vibriones of the mouth have, on various culture media, the same reaction as Koch's bacillus, would be a point of great weight, while a simple morphological similarity is of scarcely any consequence whatever.

In medical journals, particularly English journals, we continually meet with such statements as—"Comma bacilli may be found in the human mouth;" "in the intestines in various disorders;" "in certain articles of diet," etc., etc. Therefore, Koch's theory of the cause of Cholera Asiatica is entirely wrong. It is surprising that such views should ever be published by any journal. What if comma bacilli are found in the human mouth? It is the testimony of ninety-nine out of one hundred that they are *not* the comma bacilli of Koch. What if they may be found in stale cheese? The cheese spirilli have been proven to be altogether a different organism from Koch's bacillus. It matters not where comma bacilli are found, or in how great numbers. The question to be decided is whether any of these comma bacilli are identical with Koch's, and the universal verdict is that they are not. It remains to be seen whether Dr. Klein can prove his statement as to the identity of the common mouth vibriones with the cholera bacillus.

To put down all comma-shaped micro-organisms as one and the same, simply because they are curved, is no more reasonable than it would be to treat all bacilli in the same manner because they are straight, and to affirm that a particular bacillus cannot be the cause of tuberculosis, because bacilli are found in the human mouth, in the intestines, and in various articles of food.

## Figures and Tables

**Fig 1. f1:**
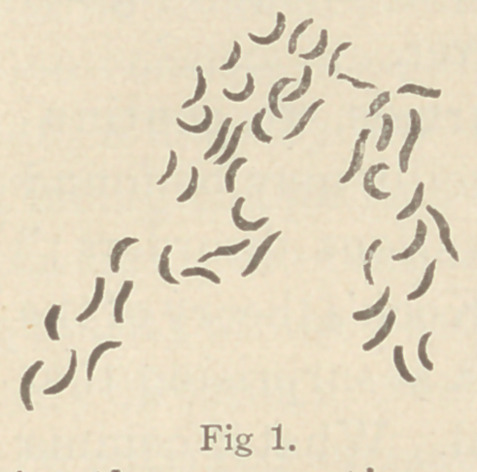


**Fig. 2. f2:**